# Poster Session II - A245 SHOTGUN METAGENOMICS IDENTIFIES RUMINCOCCUS TORQUES AND ITS METABOLIC FUNCTION AS AN IMPORTANT CONTRIBUTOR TO CROHN’S DISEASE RISK

**DOI:** 10.1093/jcag/gwaf042.245

**Published:** 2026-02-13

**Authors:** Q Li, B Bharali, F A Guevara Agudelo, S Lee, L A Dieleman, A Griffiths, H Steinhart, R Panaccione, K Jacobson, W Turpin, K Croitoru

**Affiliations:** Lunenfeld-Tanenbaum Research Institute, Toronto, ON, Canada; Lunenfeld Tanenbaum Research Institution, Sinai Health, Toronto, ON, Canada; University of Toronto, Toronto, ON, Canada; Lunenfeld Tanenbaum Research Institution, Sinai Health, Toronto, ON, Canada; University of Alberta, Edmonton, AB, Canada; The Hospital for Sick Children, Toronto, ON, Canada; Lunenfeld Tanenbaum Research Institution, Sinai Health, Toronto, ON, Canada; University of Calgary, Calgary, AB, Canada; BC Children’s Hospital, Vancouver, BC, Canada; Lunenfeld-Tanenbaum Research Institute, Toronto, ON, Canada; Lunenfeld-Tanenbaum Research Institute, Toronto, ON, Canada

## Abstract

**Background:**

Crohn’s disease (CD) incidence is rising worldwide. Prior work linked gut microbiome to CD risk, but species-level drivers and their functions before diagnosis remain poorly defined.

**Aims:**

Identify pre-diagnostic microbial species and metabolic pathways associated with future CD.

**Methods:**

Healthy first-degree relatives in the CCC-GEM cohort provided baseline stool for deep shotgun metagenomics (>30M reads/sample). Species profiles and MetaCyc pathway abundances were derived using *HUMAnN3*. Cox PH models related microbiome features to time-to-CD, adjusting for age and sex. We estimated taxon-level contributions to CD-associated pathways. High-quality *Ruminococcus torques* metagenome-assembled genomes (MAGs) were built and annotated with *eggNOG*-mapper to assess whether they encode HUMAnN-identified CD-risk MetaCyc pathways.

**Results:**

Among 955 participants, 85 developed CD during follow-up. Based on metagenomics sequencing there was a higher prevalence of *R. torques, Dorea formicigenerans, Clostridiaceae bacterium,* species that were significantly associated with future risk of CD in the 85 participants who developed CD. For metagenomics derived pathways, purine deoxyribonucleosides degradation, superpathway of purine nucleotide salvage, purine nucleobases degradation I (anaerobic), guanosine nucleotides degradation II and superpathway of glycerol degradation to 1,3-propanediol were significantly associated with increased risk of CD. Interestingly, *R. torques* contributed the most to nucleotide salvage/turnover and glycerol/glycerophospholipid metabolism pathways. Representative *R. torques* MAGs contained open reading frames consistent with glycerol utilization/glycerophospholipid remodeling and purine salvage/turnover, providing genomic support for these functional signals.

**Conclusions:**

Upon deep shotgun metagenomics analysis, pre-diagnostic enrichment of *R. torques* and its contribution to nucleotide and glycerol metabolic pathways were associated with increased risk of CD. These findings suggest that *R. torques*–mediated nucleotide turnover and lipid metabolism represent a putative functional pathway in CD pathogenesis. Ongoing work includes comparative *R. torques* pan-genome analysis between pre-CD and healthy relatives to localize risk-linked gene modules. Unraveling this pathway and its inhibition may lead to novel preventive and therapeutic interventions for CD.

**Funding Agencies:**

None

